# Supplementary material for: Projected soil carbon loss with warming in constrained Earth system models
Source: Nat Commun. 2024 Jan 2;15:102. doi: 10.1038/s41467-023-44433-2 (PMC10761705; doi:10.1038/s41467-023-44433-2)
Supplement: Supplementary file 1 — Supplementary Information [file 41467_2023_44433_MOESM1_ESM.pdf]

Supplementary Information

Projected soil carbon loss with warming in constrained Earth system models

Shuai Ren[1,2], Tao Wang[1*], Bertrand Guenet[3], Dan Liu[1], Yingfang Cao[1,2], Jinzhi Ding[1], Pete Smith[4], Shilong Piao[1,5]

[1]State Key Laboratory of Tibetan Plateau Earth System and Resources Environment (TPESRE), Institute of Tibetan Plateau Research, Chinese Academy of Sciences, Beijing, China

[2]University of Chinese Academy of Sciences, Beijing, China

[3]Laboratoire de Géologie, UMR 8538, Ecole Normale Supérieure, PSL Research University, CNRS, IPSL, Paris, France

[4]Institute of Biological and Environmental Sciences, School of Biological Sciences, University of Aberdeen, Aberdeen AB24 3UU, UK.

[5]Sino-French Institute for Earth System Science, College of Urban and environmental Sciences, Peking University, Beijing 100871, China.

Corresponding author: twang@itpcas.ac.cn

Supplementary Materials

Figures S1-S19

Tables S1-S10

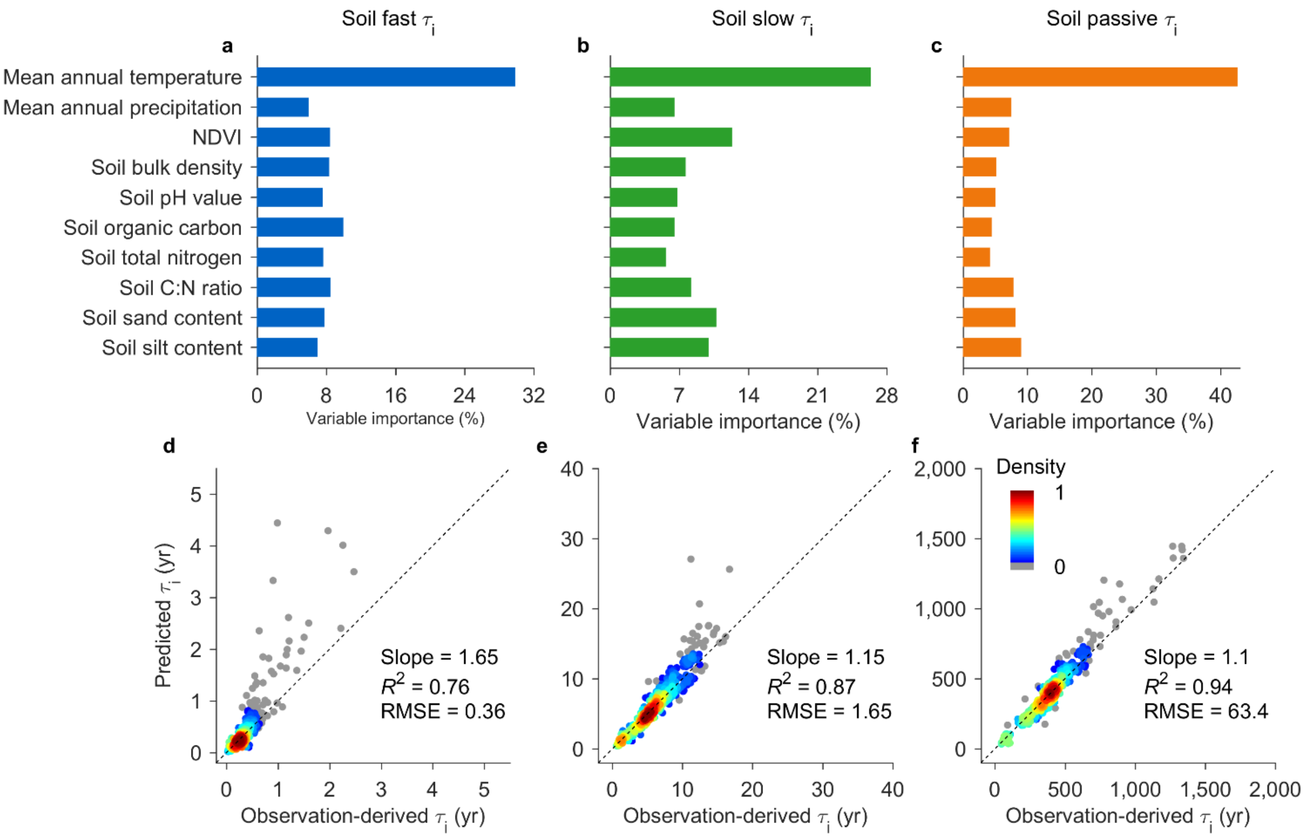

Supplementary Fig. 1 | Variables predicting intrinsic soil carbon turnover time. a–c, the relative importance of 10 different variables (climate, vegetation and soil) (see Methods) in predicting the spatial variation of intrinsic soil carbon turnover times (τ_i) for C_{fast} (**a**), C_{slow} (**b**) and C_{passive} (**c**), respectively. **d–f**, heat plots showing the relationships between predicted and inverted soil τ_i for the three carbon pools (n = 374). The dashed diagonal line is the 1:1 line between predicted and observation-derived τ_i.

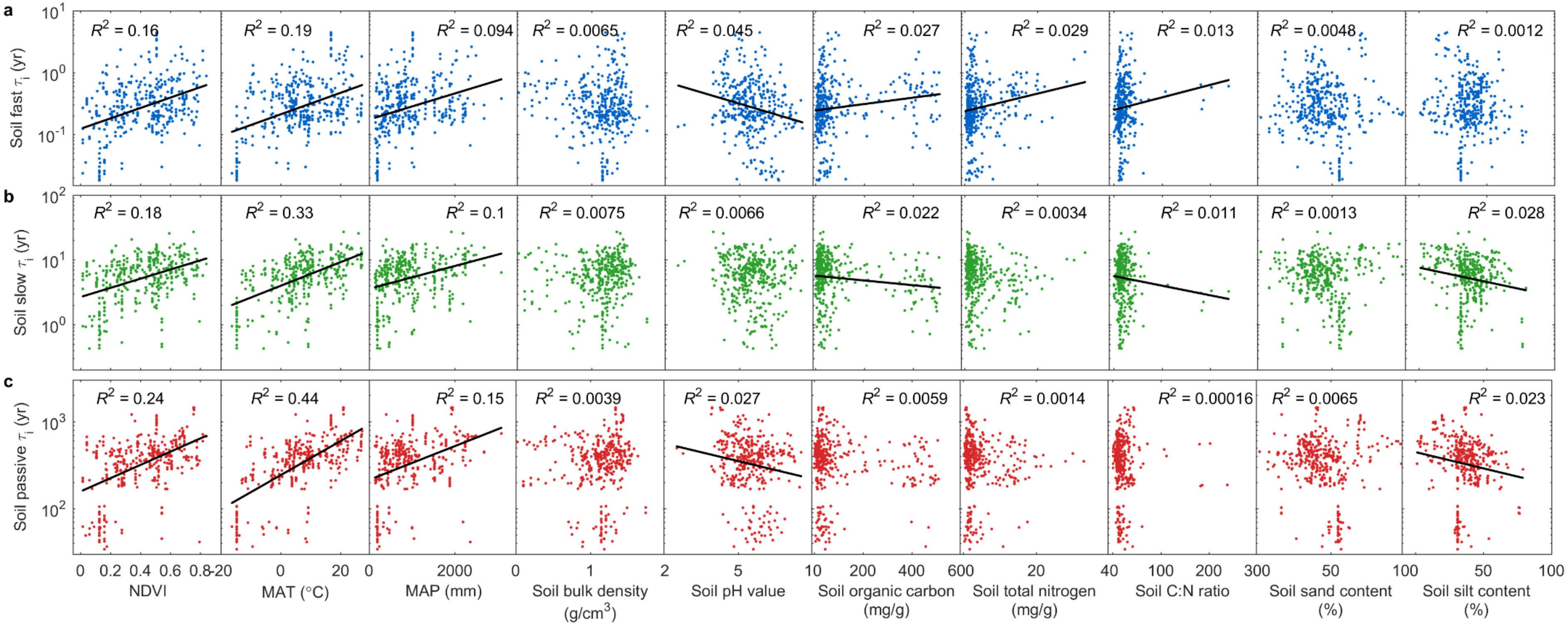

Supplementary Fig. 2 | Regression plots of intrinsic soil carbon turnover times (τ_i) with climate-, vegetation- and soil-related variables (*n* = 374) in C_{fast} (a), C_{slow} (b) and $C_{passive}$ (c). The black line denotes that the relationship is significant ($P < 0.05$). Note that the y-axis data are log-transformed.

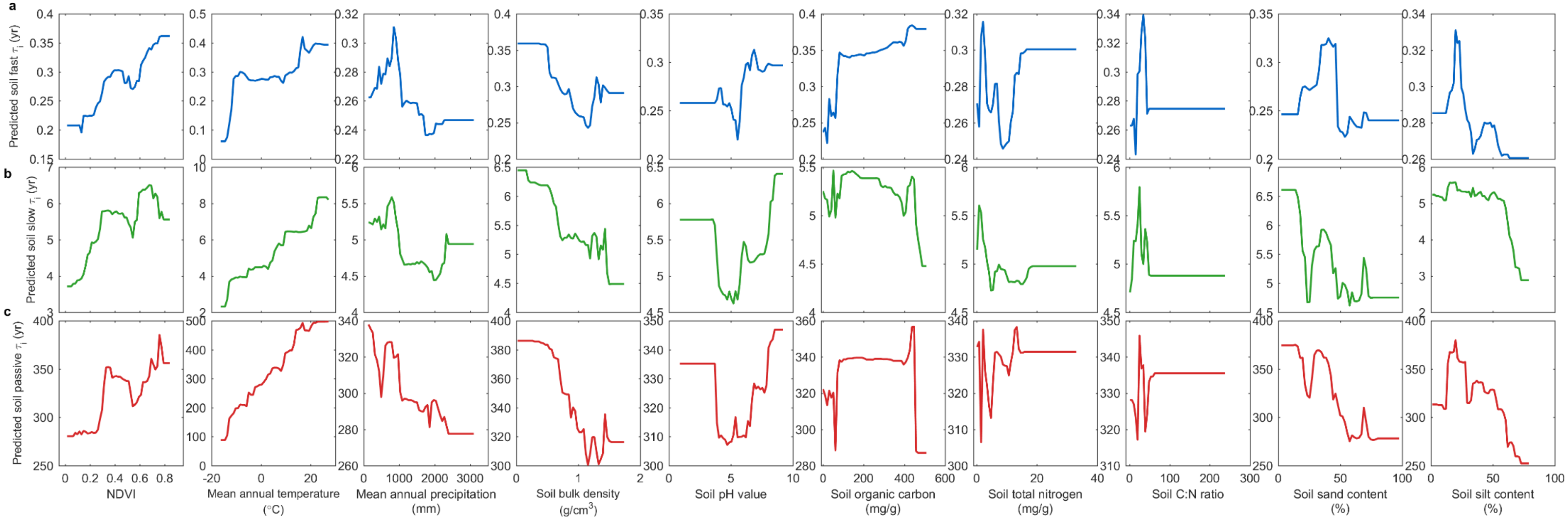

Supplementary Fig. 3 | Partial dependence plots of marginal effect of the variables on soil τ_i predictions of BRT for C_{fast} (a), C_{slow} (b) and $C_{passive}$ (c). The figures illustrate the effects of variables selected on soil τ_i predictions in BRT across a specific range of values. The small variation in predicted values usually reflects the low predictive power of the predictor.

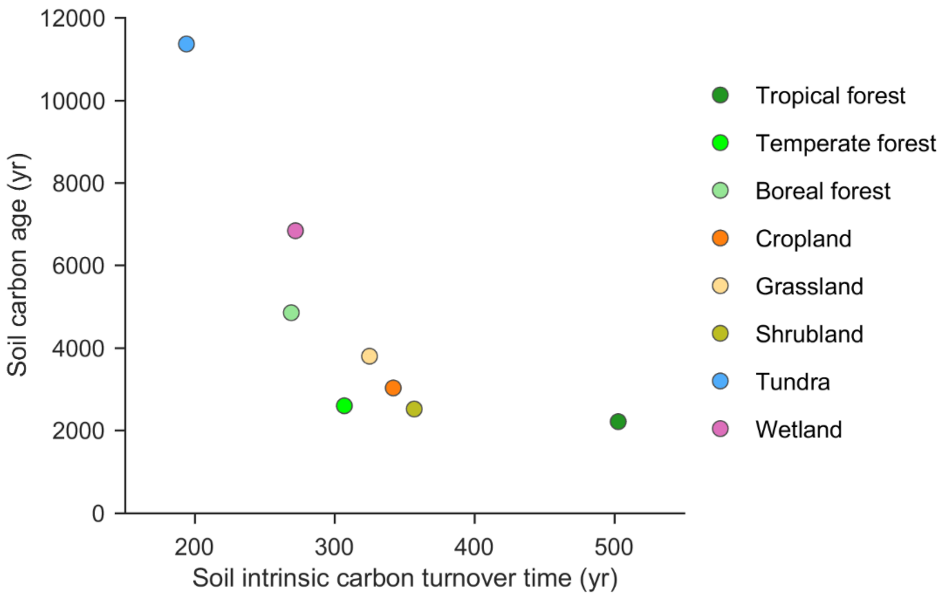

Supplementary Fig. 4 | The relationship between actual and intrinsic soil carbon turnover times at a soil depth of 1 m across different biomes. Actual soil carbon turnover time is taken from a gridded map of radiocarbon-inferred soil carbon age[1]. See Supplementary Table 1 for more details.

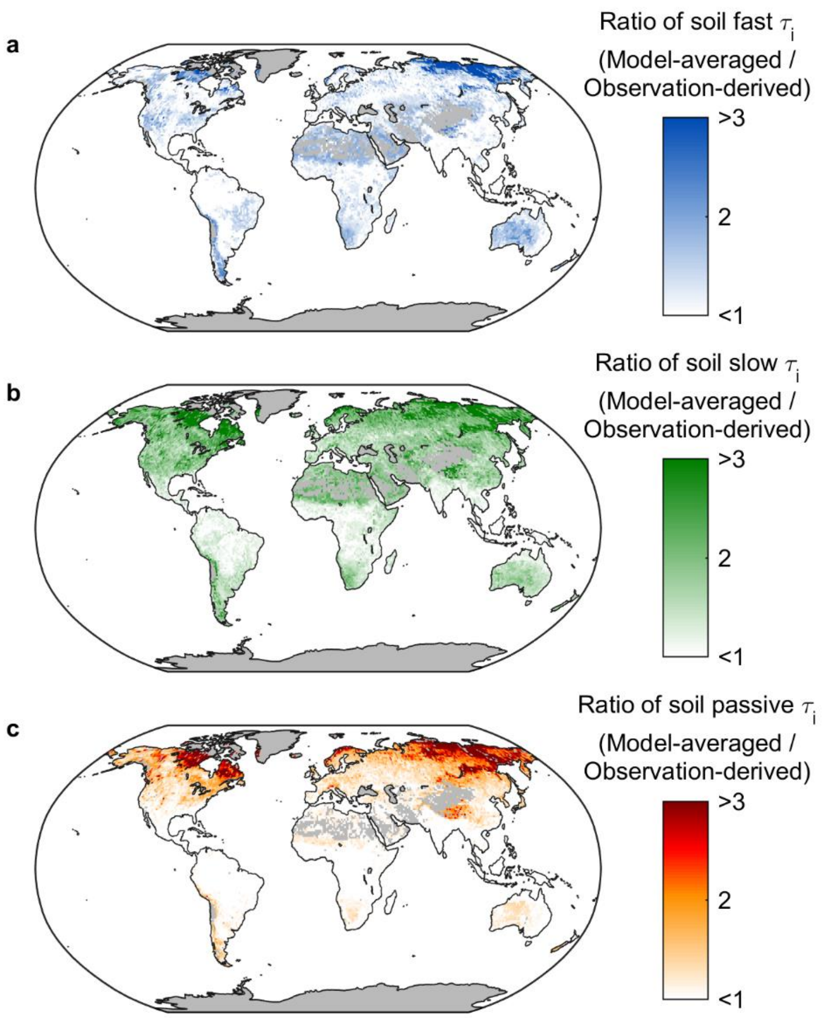

Supplementary Fig. 5 | Global distributions of the deviation of model-specified soil τ_i from observation-derived estimates for C_{fast} (a), C_{slow} (b) and $C_{passive}$ (c), respectively.

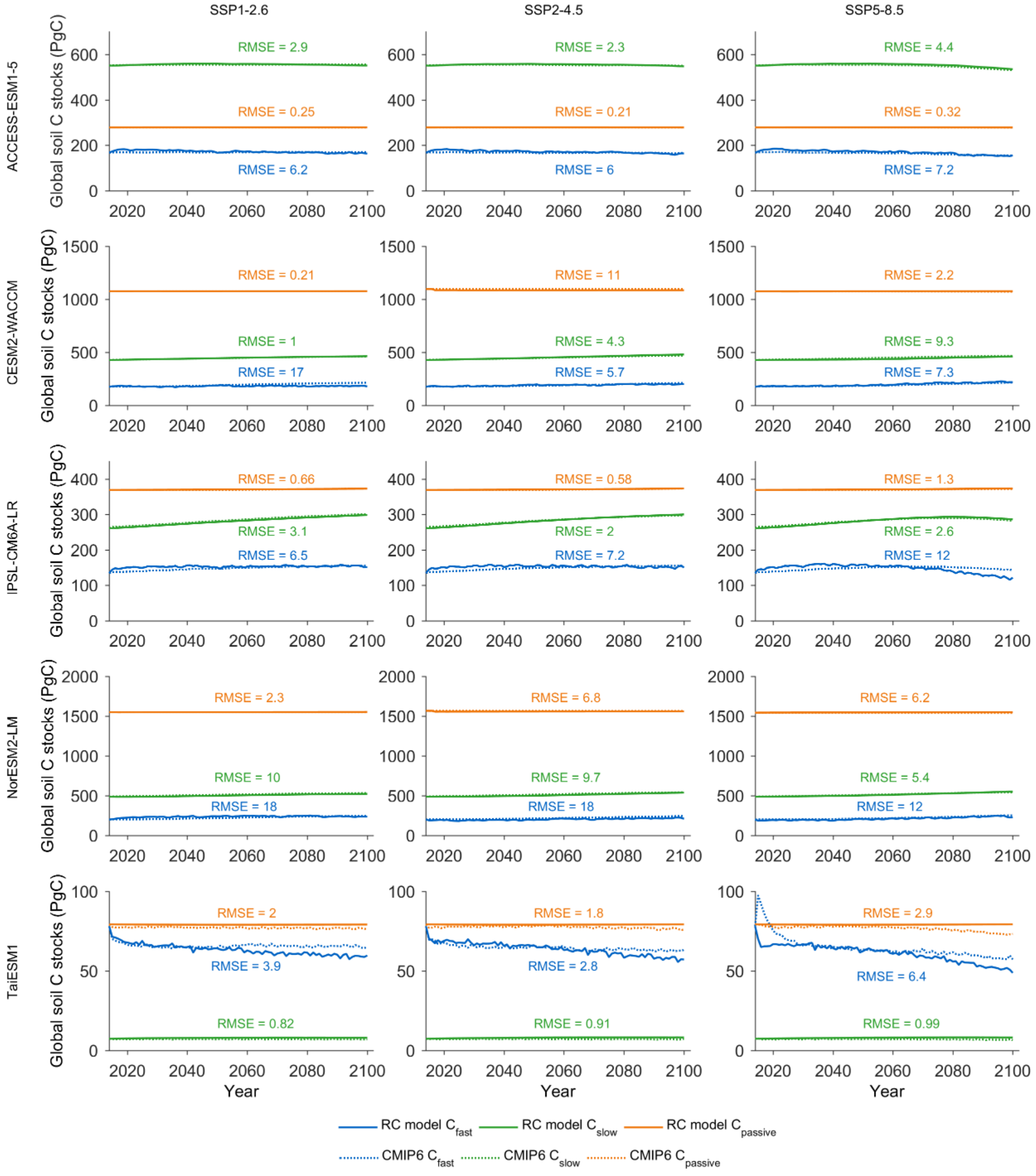

Supplementary Fig. 6 | Comparison of different global soil carbon stock projections of the three pools under SSP1-2.6, SSP2-4.5 and SSP5-8.5 scenarios. The solid and dashed lines represent projected soil carbon stocks of the three pools from the reduced-complexity model (RC model) and original CMIP6 models, respectively. RMSE, root mean square error between soil carbon stocks from the RC model and the original CMIP6 model.

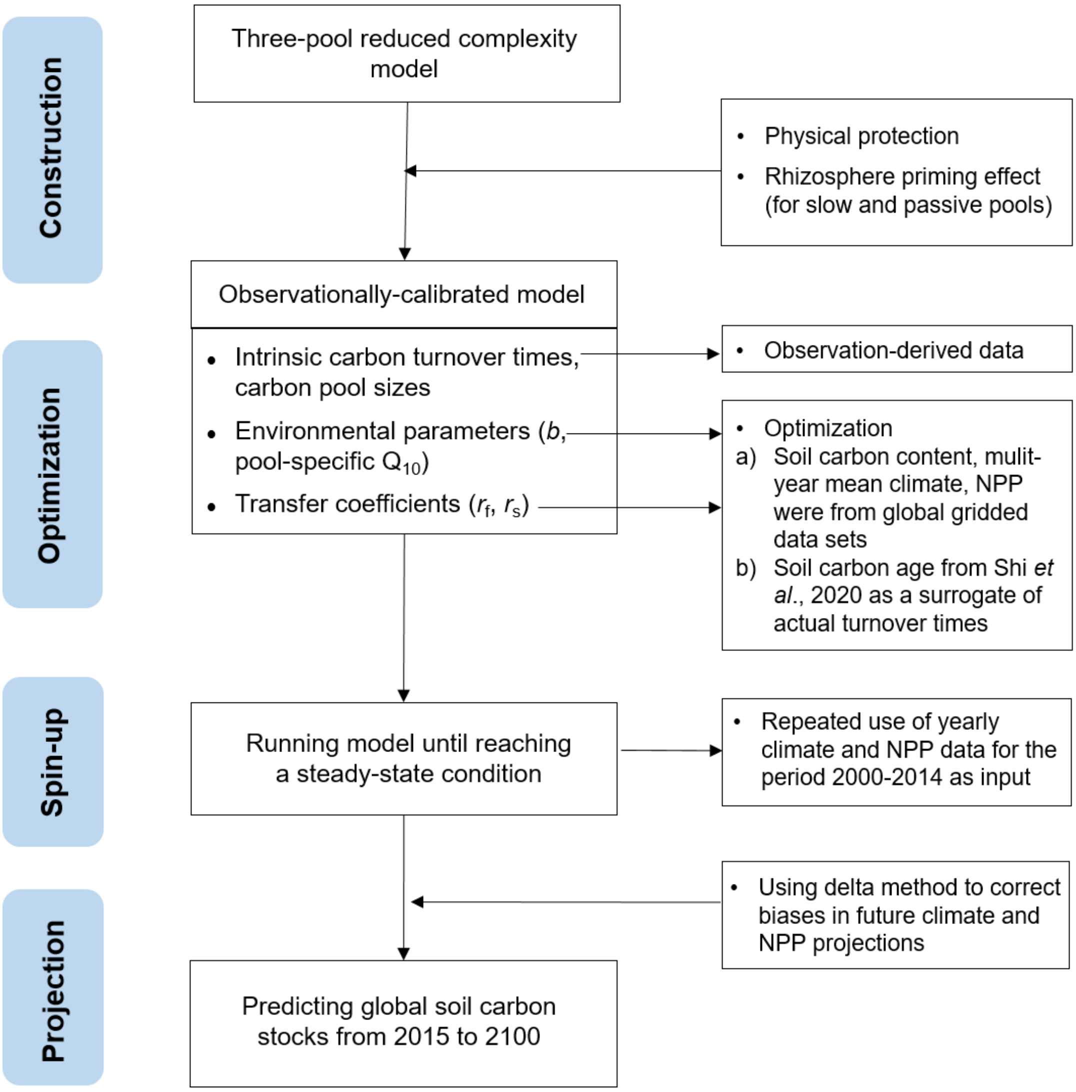

Supplementary Fig. 7 | Flowchart showing the procedure for constraining soil carbon stock changes in Earth System Models. See Methods for more details.

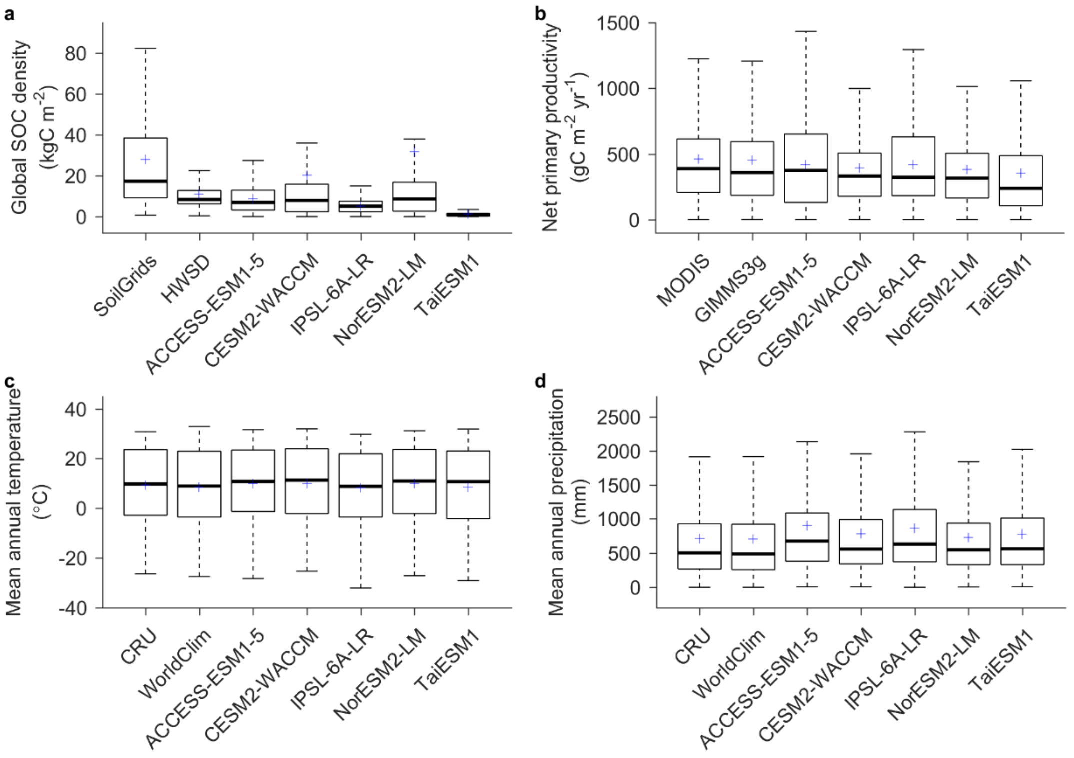

Supplementary Fig. 8 | Comparisions of SOC (a), NPP (b), MAT (c) and MAP (d) between observations and CMIP6 models during the period 2005–2014. The boxes show the median, 25th, 75th percentiles and 1.5 times the interquartile range of all grid cells. The symbol plus indicates the mean value.

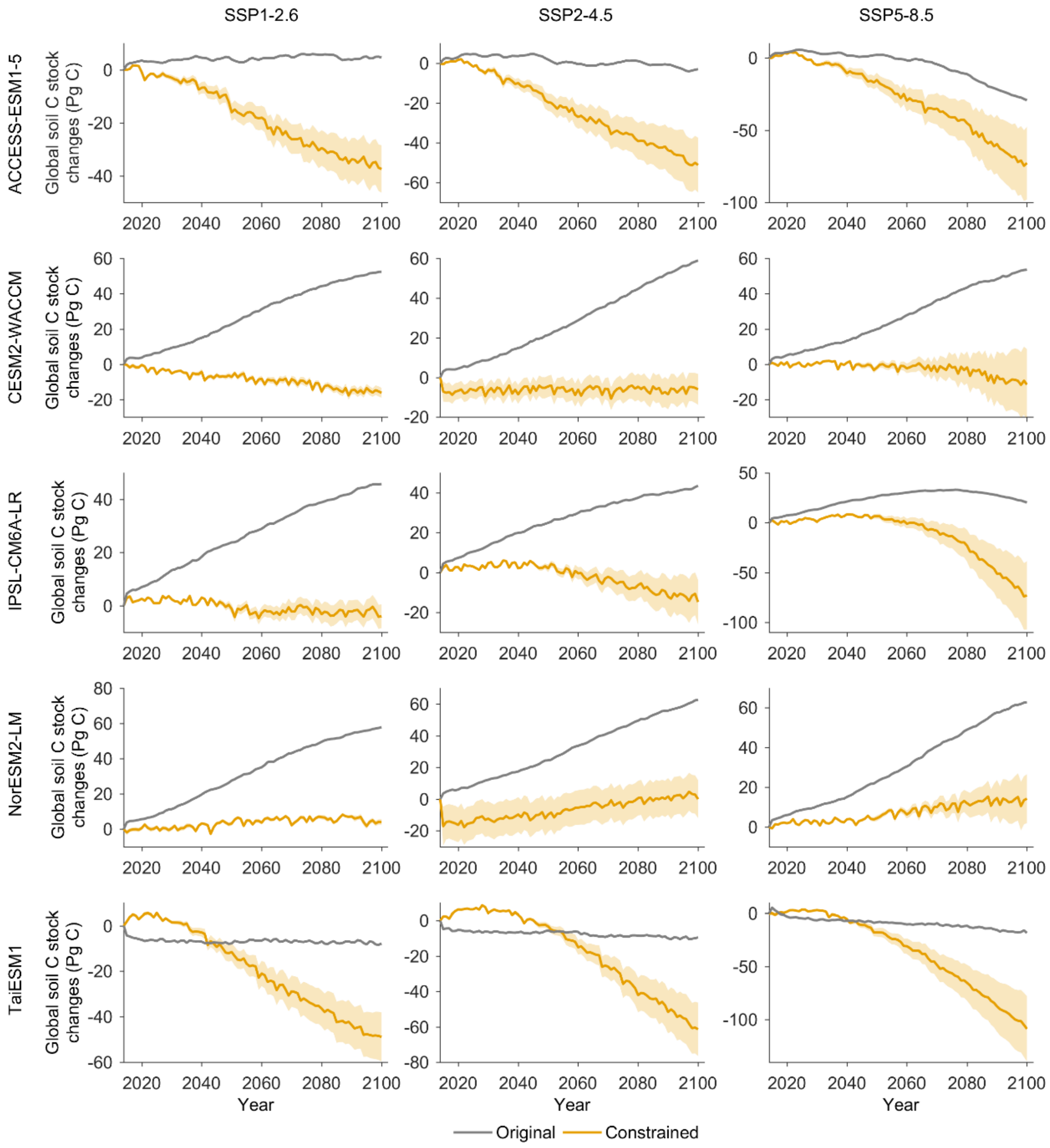

Supplementary Fig. 9 | Projected changes in global soil carbon stock from original (grey line) and constrained (orange line) Earth system models under SSP1-2.6, SSP2-4.5 and SSP5-8.5 scenarios. The shaded area represents the standard deviation of projected changes in global soil carbon stock due to the use of different combinations of soil organic carbon (SoilGrids and the Harmonized World Soil Database) and net primary productivity data sets (MODIS and Global Inventory Modeling and Mapping Studies) (see Methods).

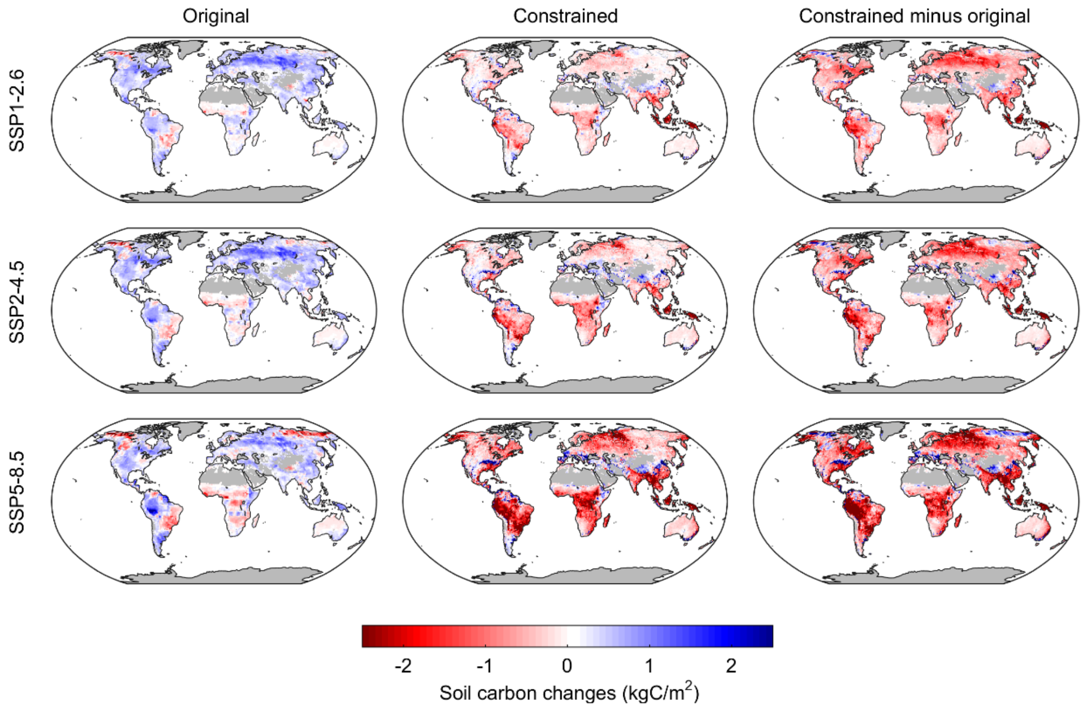

Supplementary Fig. 10 | Global distributions of projected changes in soil carbon stock between the current period (2005–2014) and the end of the century (2080–2099) from original and constrained models, and their difference under SSP1-2.6, SSP2-4.5 and SSP5-8.5 scenarios.

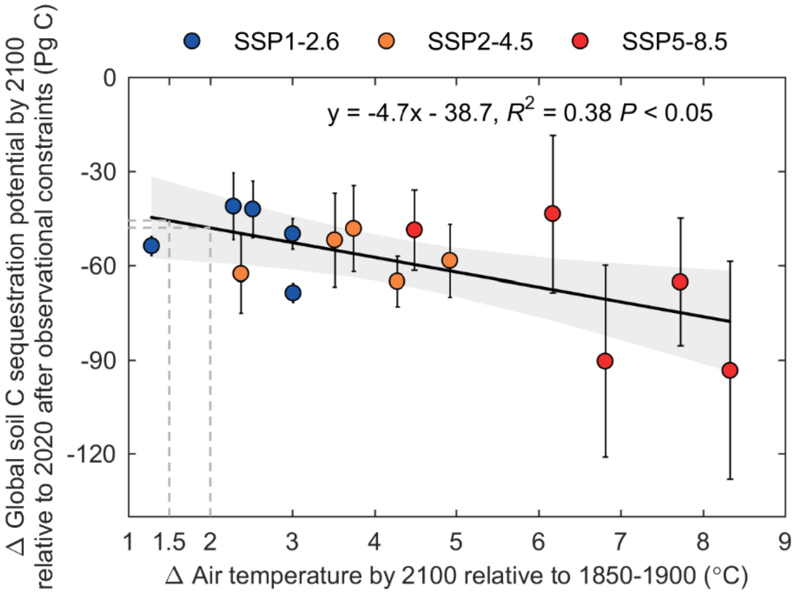

Supplementary Fig. 11 | Relationship between the magnitude of global warming and observational-constrained changes in global soil carbon stock across three different emission scenarios and five models. Changes in air temperature were calculated using the mean value of 1850–1900 as a baseline, while projected changes in global soil carbon stock by 2100 used the 2020 value as a baseline. The dashed lines indicate that under a global warming scenario of 1.5°C and 2°C by 2100, compared to the baseline period of 1850–1900, the global soil is projected to sequester 45 (33–58) and 48 (37–59) Pg less carbon than expected, respectively.

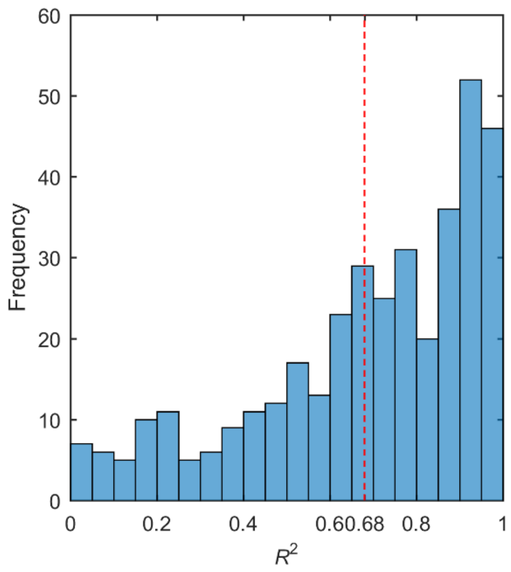

Supplementary Fig. 12 | Frequency distribution of the coefficient determination between observed soil CO_2 fluxes and those simulated by the three-pool carbon model across all experimental sites (*n* = 374). The red dashed line represents the mean value.

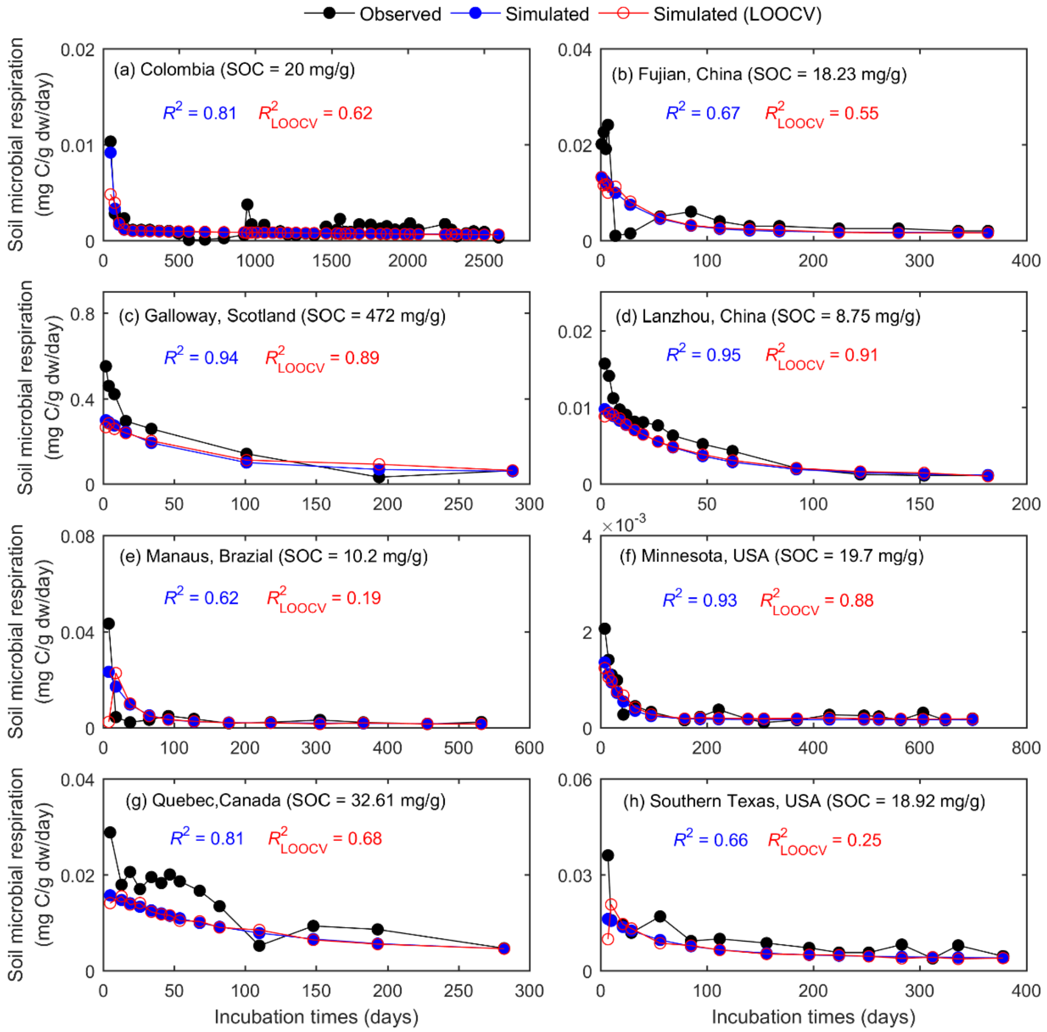

Supplementary Fig. 13 | Observed and simulated soil respiration curves for randomly selected soil incubation experiments. The incubation experiments are respectivey taken from Dharmakeerthi et al.[2] (**a**), Lei et al.[3] (**b**), Neale et al.[4] (**c**), Guo et al.[5] (**d**), Liang et al.[6] (**e**), Stewart et al.[7] (**f**), Cote et al.[8] (**g**) and Creamer et al.[9] (**h**). The black points represent observed respiration rates from soil incubation experiments, while the blue points indicate respiration rates simulated by the three-pool carbon model and the red points indicate respiration rates estimated using the Leave-One-Out Cross-Validation (LOOCV) method.

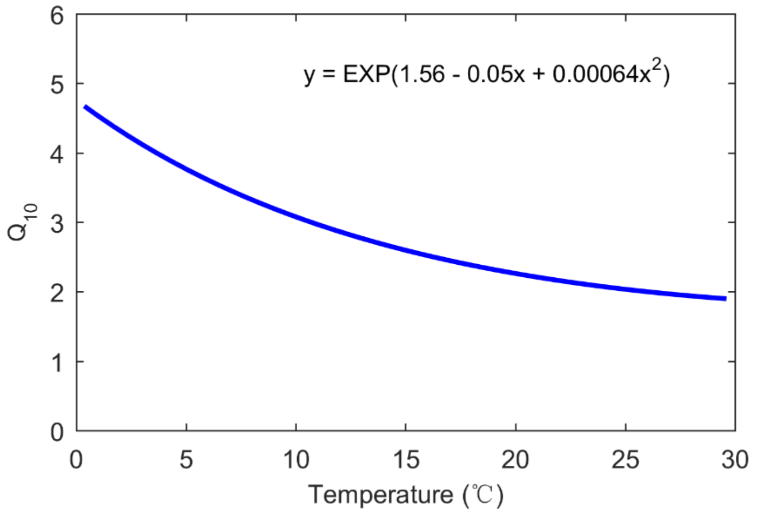

Supplementary Fig. 14 | Relationship between the temperature sensitivity of soil respiration (Q_{10}) and temperature. This relationship is derived from a synthesis analysis of Q_{10} from laboratory studies[10].

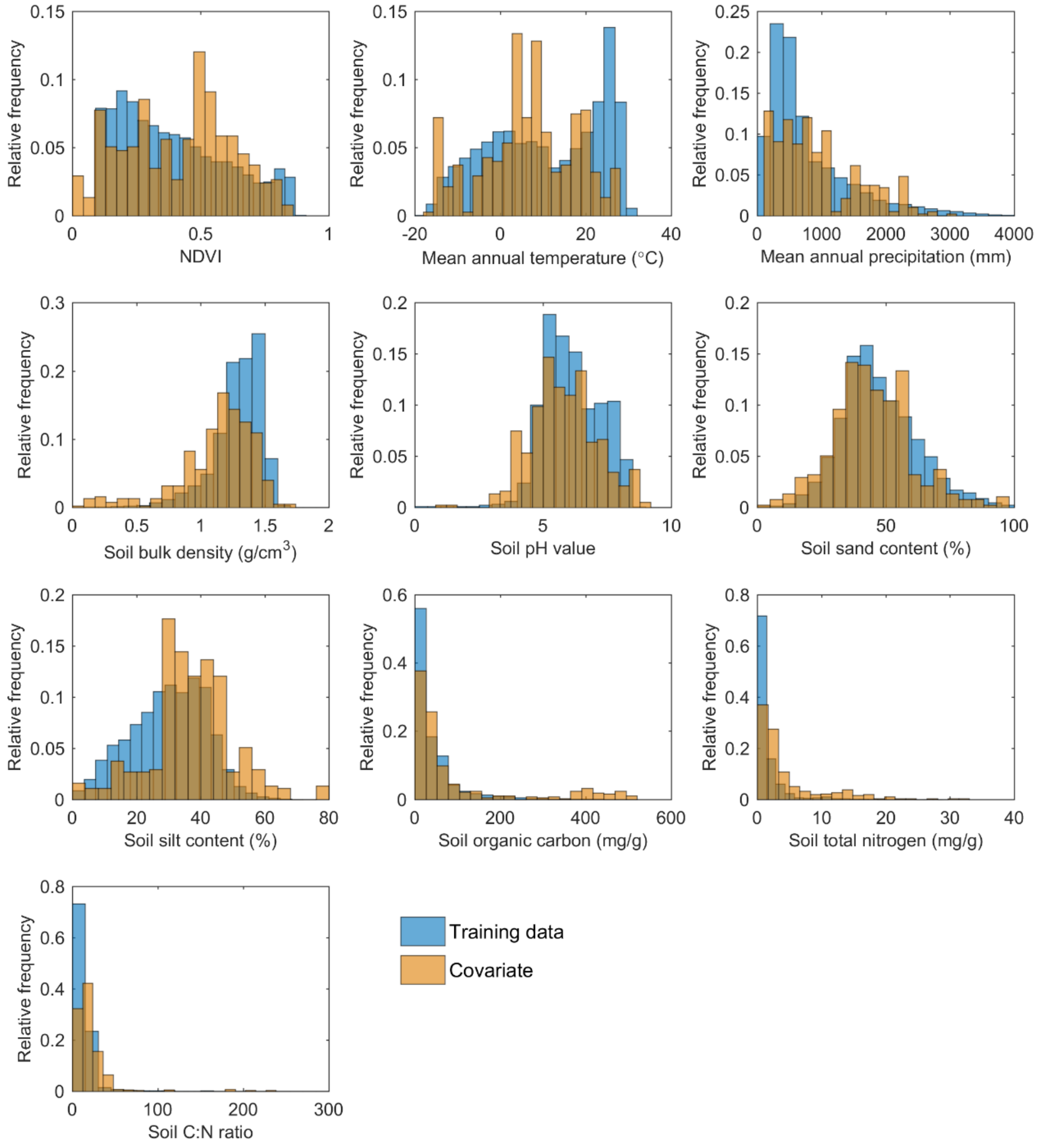

Supplementary Fig. 15 | Representation of 10 climatic and edaphic predictors in BRT upscaling. Histograms showing the distribution of predictors in our collected data (*n*=374) that were used to train BRT model and global gridded data sets that were used for upscaling. In particular, for tundra and wetland soil samples (79 in total), approximately 63% (50 of 79) of these samples were from mineral soils (carbon content < 200 mg/g; ref.[11]).

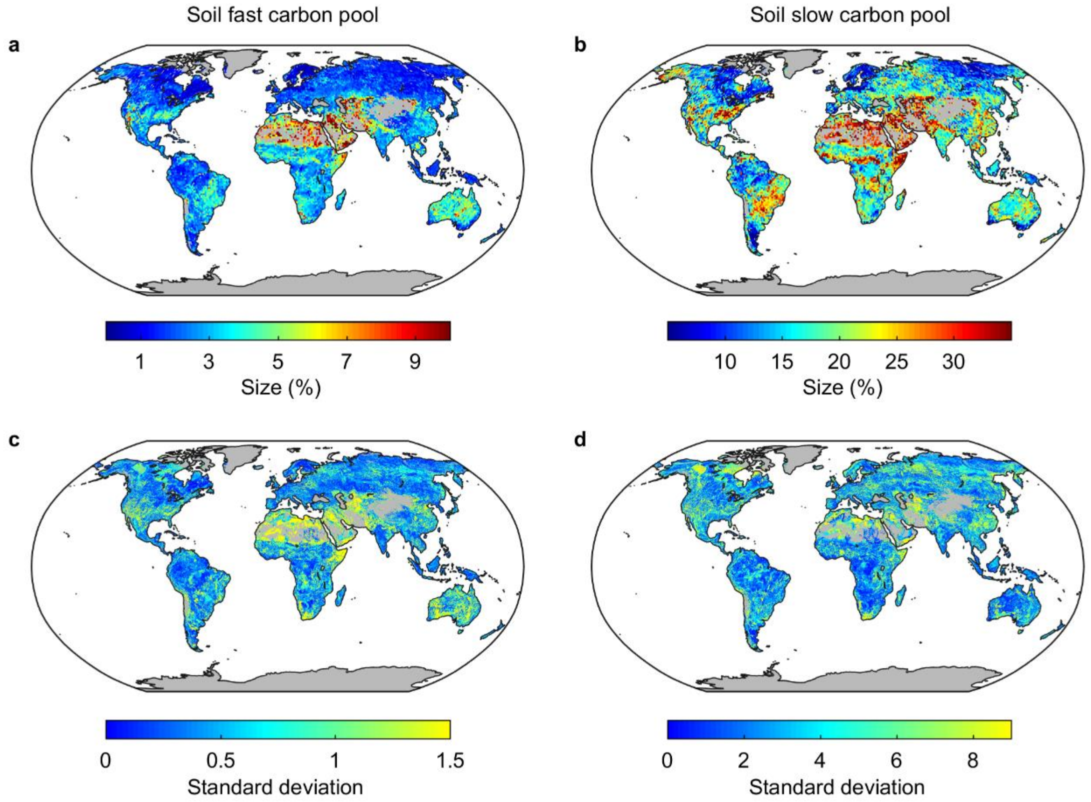

Supplementary Fig. 16 | Global distributions of carbon pool sizes for C_{fast} (a) and C_{slow} (b). These maps were upscaled from 374 incubation experiments using their empirical relationships with potential predictors in a machine learning model (R^2 = 0.87, RMSE = 1.4 for C_{fast}; R^2 = 0.88, RMSE = 7.1 for C_{slow}). **c**–**d**, the standard deviations were calculated from different combinations of climate (Climate Research Unit and WorldClim) and edaphic (Global Soil Dataset for Earth System Modeling and SoilGrids) data sets (see Methods).

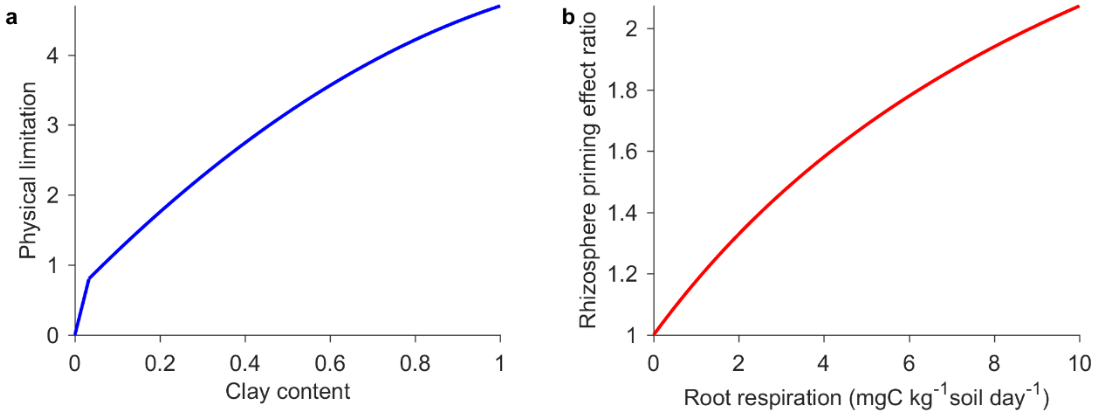

Supplementary Fig. 17 | The representation of soil physical protection and rhizosphere priming effect in refined three-pool model used to constrain model projections. The effect of soil physical protection on soil carbon dynamics is described as a function of soil clay content[12] (**a**), and the rhizosphere priming effect is described as a function of root respiration[13] (**b**).

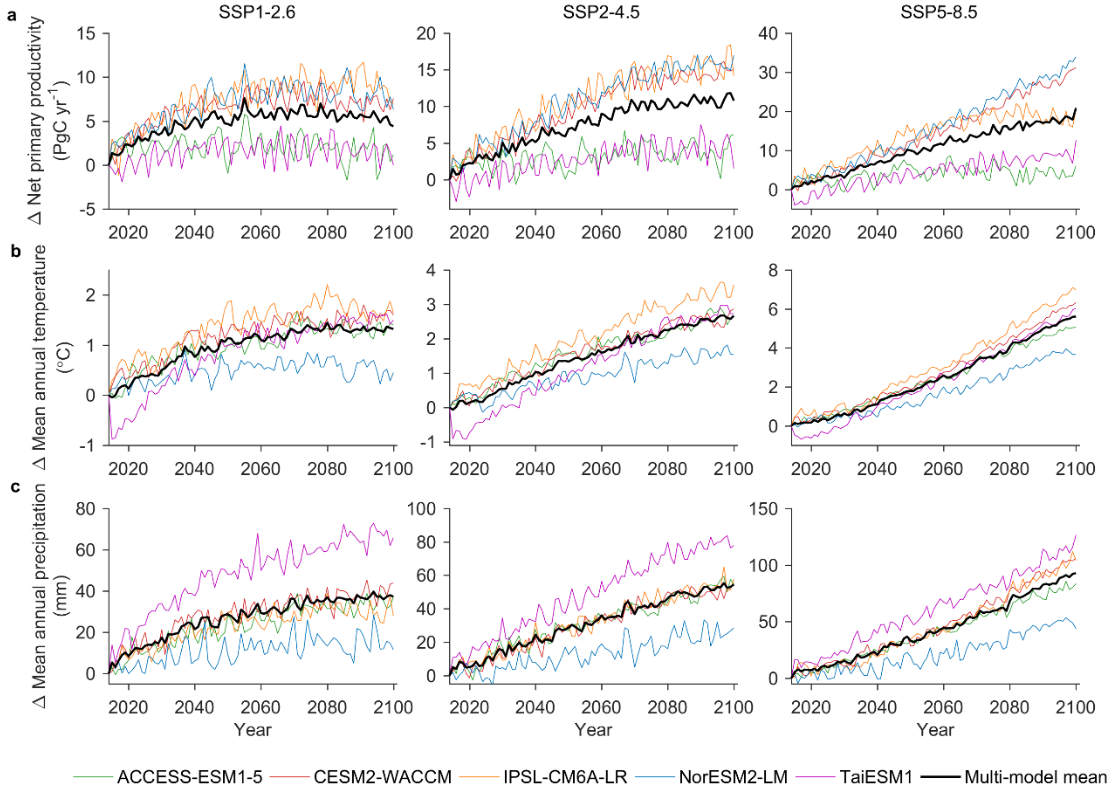

Supplementary Fig. 18 | Projected changes in net primary productivity (NPP) (a), mean annual temperature (MAT) (b) and mean annual precipitation (MAP) (c) from the five Earth system Models under SSP1-2.6, SSP2-4.5 and SSP5-8.5 scenarios.

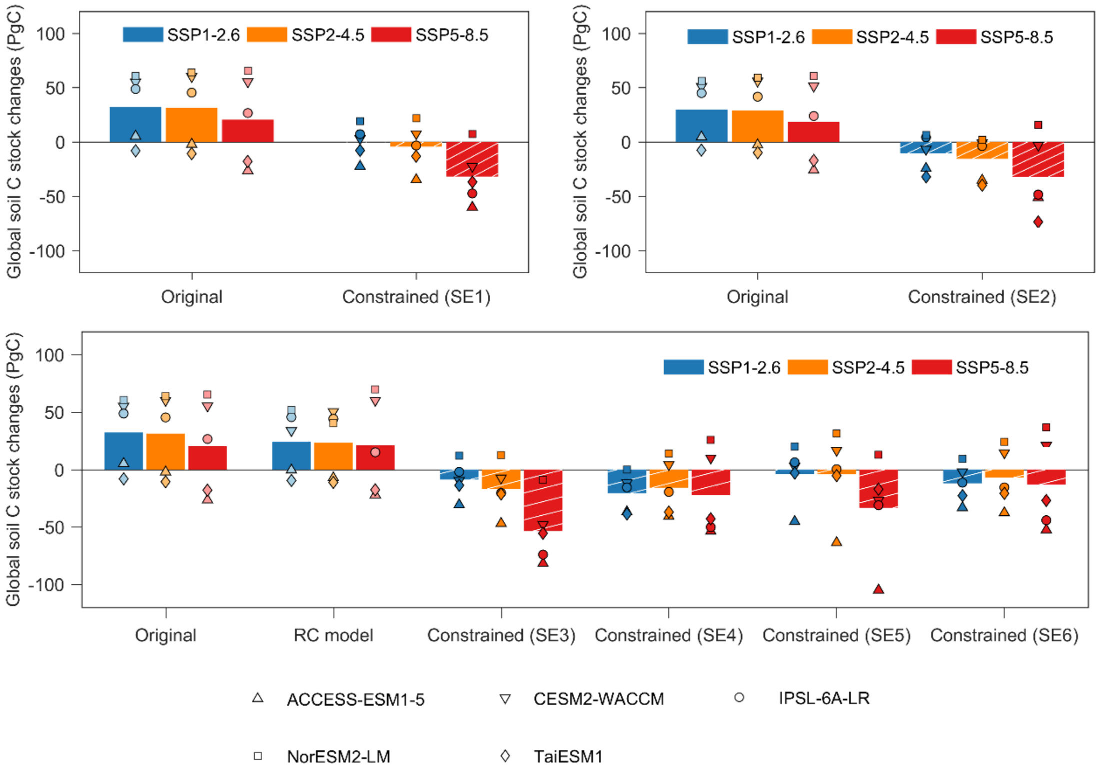

Supplementary Fig. 19 | Sensitivity experiments (SE) in this study. See Methods and Table S10 for more details.

Supplementary Table 1 | Summary statistics of observation-derived estimate of intrinsic soil carbon turnover times (τ_i) for the globe and different biomes. The biomes are based on the MODIS IGBP land classification[14]. The values are the mean and 95% confidence intervals (in parentheses). Soil carbon age (yr) data were obtained from Shi et al.[1] at a soil depth of 1 m. Ratio = soil carbon age / pool-weight τ_i.

Biome	Soil fast τ_i	Soil slow τ_i	Soil passive τ_i	Pool-weighted τ_i	Soil carbon age	Ratio
Tropical forest	0.43 (0.25, 0.73)	9.41 (7.06, 12.2)	601 (476, 742)	503 (357, 658)	2,210 (1,190, 3,341)	4.39
Temperate forest	0.33 (0.19, 0.52)	6.19 (3.68, 9.08)	380 (234, 640)	307 (182, 559)	2,594 (1,330, 4,203)	8.45
Boreal forest	0.32 (0.18, 0.51)	5.58 (4.05, 8.1)	326 (213, 455)	269 (178, 389)	4,850 (2,495, 12,197)	18
Cropland	0.33 (0.2, 0.47)	7.26 (4.74, 10.2)	434 (290, 586)	342 (239, 482)	3,027 (1,423, 4,929)	8.85
Grassland	0.28 (0.16, 0.47)	6.93 (3.63, 10.4)	418 (198, 587)	325 (156, 486)	3,795 (1,180, 11,627)	11.7
Shrubland	0.31 (0.16, 0.58)	7.8 (4.45, 12.2)	467 (277, 663)	357 (216, 556)	2,516 (1,117, 4,789)	7.05
Tundra	0.25 (0.05, 0.48)	4.48 (2.03, 6.83)	228 (77, 407)	194 (71, 346)	11,362 (3,032, 23,149)	58.6
Wetland	0.36 (0.13, 0.59)	5.68 (2.57, 12.1)	345 (122, 599)	272 (106, 460)	6,837 (1,930, 18,019)	25.1
Global	0.3 (0.11, 0.55)	6.68 (2.73, 11.2)	398 (118, 660)	316 (104, 562)	5,238 (1,213, 18,340)	16.6

Supplementary Table 2 | Model-specified soil intrinsic turnover times (τ_i; yr) of different carbon pools, Q_{10} and associated reference temperature (°C).

Model	Soil fast τ_i	Soil slow τ_i	Soil passive τ_i	Q_{10}	T_{ref}
ACCESS-ESM1-5	0.24	5	222	1.72	20
CESM2-WACCM	0.17	6.1	270	1.5	25
IPSL-6A-LR	0.149	5.48	241	2	30
NorESM2-LM	0.17	6.1	270	1.5	25
TaiESM1	0.17	6.1	270	1.5	25

Supplementary Table 3 | Information on the global data sets that were used to constrain changes in global soil carbon stock in the Earth System Models.

Data set	Source	Unit	Res	Time	Ref
Net primary productivity	MODIS MOD17A3 product	gC/m^2/yr	1km	2000–2015	Zhao et al.[15]
	Global Inventory Modeling and Mapping Studies	gC/m^2/yr	1°	1982–2011	Smith et al.[16]
Mean annual temperature	Climate Research Unit version 4.01	°C	0.5°	1901–2016	Harris et al.[17]
Mean annual precipitation	Climate Research Unit version 4.01	mm	0.5°	1901–2016	Harris et al.[17]
Soil carbon density	SoilGrids	kgC/m^2	250m	Present-day	Hengl et al.[18]
	Harmonized World Soil Database	kgC/m^2	0.05°	Present-day	Wieder et al.[19]
Soil clay content	SoilGrids	g/kg	250m	Present-day	Hengl et al.[18]
Soil carbon age	Global_age	year	0.5°	Present-day	Shi et al.[1]
Soil pool-specific τ_i	Upscaled	year	0.1°	Present-day	-
Soil pool-specific carbon fraction	Upscaled	%	0.1°	Present-day	-

Supplementary Table 4 | Description of the parameters in the three-pool carbon model.

Parameter	Description	Unit	Lower limit	Upper limit
f_{fast}	Fraction of C_{fast}	-	0	0.2
f_{slow}	Fraction of C_{slow}	-	0	1
k_{fast} ($\times 10^{-3}$)	Intrinsic decay rate of C_{fast}	d^{-1}	0.274	54.795
k_{slow} ($\times 10^{-3}$)	Intrinsic decay rate of C_{slow}	d^{-1}	0.137	2.74
$k_{passive}$ ($\times 10^{-5}$)	Intrinsic decay rate of $C_{passive}$	d^{-1}	0.274	2.74

a. the priors were set according to previous studies[11,20,21].

Supplementary Table 5 | Information on predictors that were used to upscale the site-level intrinsic soil carbon turnover times (τ_i) and carbon pool sizes to the global level. If the values of predictors were not available in published studies, we extract them from the following gridded data.

Predictor	Unit	Type	Data reported in studies	Global gridded data
Mean annual temperature	°C	Climate	194	Climate Research Unit version 4.01; WorldClim version 2.0
Mean annual precipitation	mm	Climate	194	Climate Research Unit version 4.01; WorldClim version 2.0
NDVI	-	Vegetation	0	Moderate Resolution Imaging Spectroradiometer product (MODIS)
Bulk density	g/cm^3	Soil	70	Global Soil Dataset for Earth System Modeling (GSDE)[22]; SoilGrids
pH	-	Soil	239	GSDE; SoilGrids
Organic carbon	mg/g	Soil	374	GSDE; SoilGrids
Total nitrogen	mg/g	Soil	302	GSDE
C:N ratio	-	Soil	302	GSDE
Sand content	%	Soil	70	GSDE; SoilGrids
Silt content	%	Soil	70	GSDE; SoilGrids

Supplementary Table 6 | The best hypermeter combination of a boosted regression trees modeling. A list of hypermeters was selected using ten-fold cross-validation, varying in (1) shrinkage (0.01 or 0.1), (2) interaction.depth (1 to 9, step=2), (3) n.minobsinnode (5 to 20, step=5) and (4) n.trees (50 to 1000, step=50). The data were log10 transformed before analysis.

Variables	shrinkage	interaction.depth	n.minobsinnode	n.trees	$RMSE_{cv}$	R^2_{cv}
$\tau_{i,\ fast}$	0.01	9	10	700	0.32	0.45
$\tau_{i,\ slow}$	0.01	9	5	1000	0.22	0.6
$\tau_{i,\ passive}$	0.01	9	5	900	0.16	0.71
f_{fast}	0.01	9	5	1000	0.36	0.59
f_{slow}	0.01	9	5	1000	0.32	0.71

Supplementary Table 7 | A summary of the five CMIP6 models. These models were selected since they provided future soil carbon dynamics for the three different pools (*cFast, cMedium* and *cSlow*). The data were projected to 1 degree before analysis.

Model	Institution	Resolution (longitude×latitude)	Reference
ACCESS-ESM1-5	Commonwealth Scientific and Industrial Research Organisation, Australia	192×145	Ziehn et al.(*23*)
CESM2-WACCM	National Center for Atmospheric Research, USA	288×192	Lawrence et al.(*24*)
IPSL-6A-LR[a]	Institut Pierre Simon Laplace, France	144×143	Boucher et al.(*25*)
NorESM2-LM	Norwegian Climate Centre, Norway	144×96	Seland et al.(*26*)
TaiESM1	Research Center for Environmental Changes, China	288×192	Wang et al.(*27*)

a. Noting that in ORCHIDEE, the intrinsic turnover of the fast pool is partially controlled by the clay content, but it is far from being the best representation.

Supplementary Table 8 | Description of the parameters in the reduced complexity model.

Parameter	Description	Unit	Lower limit	Upper limit
r_f	Carbon flows from C_{fast} to C_{slow}	-	0	0.5
r_s	Carbon flows from C_{slow} to $C_{passive}$	-	0	0.5
b	Precipitation coefficient	-	0	3

a. The priors were set according to our observation-driven estimates and previous studies[28-30].

Supplementary Table 9 | Description of the parameters in the refined model.

Parameter	Description	Lower limit	Upper limit	Global mean (constrained)
r_f	Carbon flows from C_{fast} to C_{slow}	0	0.5	0.21 ± 0.03
r_s	Carbon flows from C_{slow} to $C_{passive}$	0	0.5	0.08 ± 0.003
b	Precipitation coefficient	0	3	0.53 ± 0.002
$Q_{10, fast}$	Temperature sensitivity of C_{fast} at 15℃	0	3	1.88 ± 0.21
$Q_{10, slow}$	Temperature sensitivity of C_{slow} at 15°C	0	5	2.85 ± 0.02
$Q_{10, passive}$	Temperature sensitivity of $C_{passive}$ at 15°C	0	7	3.77 ± 0.16

a. The priors of Q_{10} were set according to previous studies[31,32].

Supplementary Table 10 | Description of sensitivity experiments (SE) in this study.

Options	Global data-driven soil τ_i estimates		Model structure		Model inputs (NPP, MAT and MAP)		Q_{10}		τ_i of C_{fast} and C_{slow}		τ_i of $C_{passive}$	
	Using empirical Q_{10}-temperature function to scale τ_i to 15°C at the site level	Using Arrhenius function to scale τ_i to 15°C at the site level	Climate, physical protection and priming controls on soil carbon turnover	Climate controls on soil carbon turnover	Satellite observations	Original ESMs	Calibrated against observations	ESM's own values	Data-driven values	ESM's own values	Data-driven values	ESM's own values
Default	√		√		√		√		√		√	
SE1		√	√		√		√		√		√	
SE2	√		√		√		√		√			√
SE3	√			√		√	√		√		√	
SE4	√			√		√		√	√		√	
SE5	√			√		√	√			√		√
SE6	√			√		√		√	√			√

References

1. Shi, Z., et al. The age distribution of global soil carbon inferred from radiocarbon measurements. *Nat. Geosci.* **13**, 555-559 (2020).
2. Dharmakeerthi, R.S., Hanley, K., Whitman, T., Woolf, D. & Lehmann, J. Organic carbon dynamics in soils with pyrogenic organic matter that received plant residue additions over seven years. *Soil Biol. Biochem.* **88**, 268-274 (2015).
3. Lei, H., et al. Impacts of Biochar Input on Soil Carbon Emission and Microbial Community Composition in Cunninghamia lanceolata Plantation. *Sci. Silvae Sin.* **52**, 37-44 (2016).
4. Neale, S.P., Shah, Z. & Adams, W.A. Changes in microbial biomass and nitrogen turnover in acidic organic soils following liming. *Soil Biol. Biochem.* **29**, 1463-1474 (1997).
5. Guo, D., Wang, J., Fu, H., Wen, H. & Luo, Y. Cropland has higher soil carbon residence time than grassland in the subsurface layer on the Loess Plateau, China. *Soil & Tillage Research* **174**, 130-138 (2017).
6. Liang, B., et al. Black carbon affects the cycling of non-black carbon in soil. *Org. Geochem.* **41**, 206-213 (2010).
7. Stewart, C.E., Zheng, J., Botte, J. & Cotrufo, M.F. Co-generated fast pyrolysis biochar mitigates green-house gas emissions and increases carbon sequestration in temperate soils. *GCB Bioenergy* **5**, 153-164 (2013).
8. Cote, L., Brown, S., Pare, D., Fyles, J. & Bauhus, J. Dynamics of carbon acid nitrogen mineralization in relation to stand type, stand age and soil texture in the boreal mixedwood. *Soil Biol. Biochem.* **32**, 1079-1090 (2000).
9. Creamer, C.A., Filley, T.R., Boutton, T.W., Oleynik, S. & Kantola, I.B. Controls on soil carbon accumulation during woody plant encroachment: Evidence from

physical fractionation, soil respiration, and delta C-13 of respired CO2. *Soil Biol. Biochem.* **43**, 1678-1687 (2011).

10. Hamdi, S., Moyano, F., Sall, S., Bernoux, M. & Chevallier, T. Synthesis analysis of the temperature sensitivity of soil respiration from laboratory studies in relation to incubation methods and soil conditions. *Soil Biol. Biochem.* **58**, 115-126 (2013).
11. Schaedel, C., et al. Circumpolar assessment of permafrost C quality and its vulnerability over time using long-term incubation data. *Glob. Change Biol.* **20**, 641-652 (2014).
12. Sulman, B.N., Phillips, R.P., Oishi, A.C., Shevliakova, E. & Pacala, S.W. Microbe-driven turnover offsets mineral-mediated storage of soil carbon under elevated CO2. *Nat. Clim. Change* **4**, 1099-1102 (2014).
13. Keuper, F., et al. Carbon loss from northern circumpolar permafrost soils amplified by rhizosphere priming. *Nat. Geosci.* **13**, 560-565 (2020).
14. Friedl, M.A., et al. MODIS Collection 5 global land cover: Algorithm refinements and characterization of new datasets. *Remote Sens. Environ.* **114**, 168-182 (2010).
15. Zhao, M. & Running, S.W. Drought-Induced Reduction in Global Terrestrial Net Primary Production from 2000 Through 2009. *Science* **329**, 940-943 (2010).
16. Smith, W.K., et al. Large divergence of satellite and Earth system model estimates of global terrestrial CO_2 fertilization. *Nat. Clim. Change* **6**, 306-310 (2016).
17. Harris, I., Osborn, T.J., Jones, P. & Lister, D. Version 4 of the CRU TS monthly high-resolution gridded multivariate climate dataset. *Scientific data* **7**, 1-18 (2020).
18. Hengl, T., et al. SoilGrids250m: Global gridded soil information based on machine learning. *PLoS One* **12**(2017).
19. Wieder, W., Boehnert, J., Bonan, G. & Langseth, M. Regridded harmonized world

soil database v1. 2. ORNL DAAC (2014).

20. Schaedel, C., Luo, Y., Evans, R.D., Fei, S. & Schaeffer, S.M. Separating soil CO2 efflux into C-pool-specific decay rates via inverse analysis of soil incubation data. *Oecologia* **171**, 721-732 (2013).

21. Craine, J.M., Fierer, N. & McLauchlan, K.K. Widespread coupling between the rate and temperature sensitivity of organic matter decay. *Nat. Geosci.* **3**, 854-857 (2010).

22. Shangguan, W., Dai, Y., Duan, Q., Liu, B. & Yuan, H. A global soil data set for earth system modeling. *J. Adv. Model. Earth Syst.* **6**, 249-263 (2014).

23. Ziehn, T., et al. The Australian earth system model: ACCESS-ESM1. 5. *J. South. Hemisph. Earth Syst. Sci.* **70**, 193-214 (2020).

24. Lawrence, D.M., et al. The Community Land Model version 5: Description of new features, benchmarking, and impact of forcing uncertainty. *J. Adv. Model. Earth Syst.* **11**, 4245-4287 (2019).

25. Boucher, O., et al. Presentation and evaluation of the IPSL-CM6A-LR climate model. *J. Adv. Model. Earth Syst.* **12**, e2019MS002010 (2020).

26. Seland, Ø., et al. Overview of the Norwegian Earth System Model (NorESM2) and key climate response of CMIP6 DECK, historical, and scenario simulations. *Geosci. Model Dev* **13**, 6165-6200 (2020).

27. Wang, Y.C., et al. Performance of the Taiwan Earth System Model in Simulating Climate Variability Compared With Observations and CMIP6 Model Simulations. *J. Adv. Model. Earth Syst.* **13**, e2020MS002353 (2021).

28. He, Y., et al. Radiocarbon constraints imply reduced carbon uptake by soils during the 21st century. *Science* **353**, 1419-1424 (2016).

29. Shi, Z., Crowell, S., Luo, Y. & Moore, B., III. Model structures amplify

uncertainty in predicted soil carbon responses to climate change. *Nat. Commun.* **9**(2018).

30. Todd-Brown, K.E.O., et al. Causes of variation in soil carbon simulations from CMIP5 Earth system models and comparison with observations. *Biogeosciences* **10**, 1717-1736 (2013).

31. Liang, J., et al. Methods for estimating temperature sensitivity of soil organic matter based on incubation data: A comparative evaluation. *Soil Biol. Biochem.* **80**, 127-135 (2015).

32. Ren, S., et al. Higher Temperature Sensitivity of Soil C Release to Atmosphere From Northern Permafrost Soils as Indicated by a Meta-Analysis. *Glob. Biogeochem. Cycles* **34**(2020).
