# Supplementary material for: Projected soil carbon loss with warming in constrained Earth system models
Source: Nat Commun. 2024 Jan 2;15:102. doi: 10.1038/s41467-023-44433-2 (PMC10761705; doi:10.1038/s41467-023-44433-2)
Supplement: Supplementary file 3 — Reporting Summary [file 41467_2023_44433_MOESM3_ESM.pdf]

nature portfolio

Corresponding author(s): Tao Wang
Last updated by author(s): Dec 8, 2023

Reporting Summary

Nature Portfolio wishes to improve the reproducibility of the work that we publish. This form provides structure for consistency and transparency in reporting. For further information on Nature Portfolio policies, see our Editorial Policies and the Editorial Policy Checklist.

Statistics

For all statistical analyses, confirm that the following items are present in the figure legend, table legend, main text, or Methods section.

n/a	Confirmed	
☐	☒	The exact sample size (*n*) for each experimental group/condition, given as a discrete number and unit of measurement
☒	☐	A statement on whether measurements were taken from distinct samples or whether the same sample was measured repeatedly
☒	☐	The statistical test(s) used AND whether they are one- or two-sided<br>*Only common tests should be described solely by name; describe more complex techniques in the Methods section.*
☐	☒	A description of all covariates tested
☒	☐	A description of any assumptions or corrections, such as tests of normality and adjustment for multiple comparisons
☐	☒	A full description of the statistical parameters including central tendency (e.g. means) or other basic estimates (e.g. regression coefficient) AND variation (e.g. standard deviation) or associated estimates of uncertainty (e.g. confidence intervals)
☐	☒	For null hypothesis testing, the test statistic (e.g. *F*, *t*, *r*) with confidence intervals, effect sizes, degrees of freedom and *P* value noted<br>*Give P values as exact values whenever suitable.*
☐	☒	For Bayesian analysis, information on the choice of priors and Markov chain Monte Carlo settings
☒	☐	For hierarchical and complex designs, identification of the appropriate level for tests and full reporting of outcomes
☒	☐	Estimates of effect sizes (e.g. Cohen's *d*, Pearson's *r*), indicating how they were calculated

Our web collection on statistics for biologists contains articles on many of the points above.

Software and code

Policy information about availability of computer code

Data collection	Data were taken directly from tables or extracted from figures using the GetData (v.2.25) software.
Data analysis	Data analysis was carried out using R v.4.0.5 and MATLAB R2016a. The code used in this study is available at the Figshare data repository (https://figshare.com/s/0febe56304920b8536f0)

For manuscripts utilizing custom algorithms or software that are central to the research but not yet described in published literature, software must be made available to editors and reviewers. We strongly encourage code deposition in a community repository (e.g. GitHub). See the Nature Portfolio guidelines for submitting code & software for further information.

Data

Policy information about availability of data

All manuscripts must include a data availability statement. This statement should provide the following information, where applicable:

- Accession codes, unique identifiers, or web links for publicly available datasets
- A description of any restrictions on data availability
- For clinical datasets or third party data, please ensure that the statement adheres to our policy

The collected metadata and gridded maps of soil τi and fractions of different carbon pools can be found at the Figshare data repository (https://doi.org/10.6084/m9.figshare.19641759.v1)

Research involving human participants, their data, or biological material

Policy information about studies with human participants or human data. See also policy information about sex, gender (identity/presentation), and sexual orientation and race, ethnicity and racism.

Reporting on sex and gender	N/A
Reporting on race, ethnicity, or other socially relevant groupings	N/A
Population characteristics	N/A
Recruitment	N/A
Ethics oversight	N/A

Note that full information on the approval of the study protocol must also be provided in the manuscript.

Field-specific reporting

Please select the one below that is the best fit for your research. If you are not sure, read the appropriate sections before making your selection.

☐ Life sciences ☐ Behavioural & social sciences ☒ Ecological, evolutionary & environmental sciences

For a reference copy of the document with all sections, see nature.com/documents/nr-reporting-summary-flat.pdf

Ecological, evolutionary & environmental sciences study design

All studies must disclose on these points even when the disclosure is negative.

Study description	In this study, we assembled a raw dataset of soil CO2 fluxes from long-term soil incubation experiments (n=374), and used a machine learning approach to generate a spatially-explicit understanding of pool-specific intrinsic soil carbon turnover (τi) across the globe. Using the global data-driven soil τi, we then developed a observationally-calibrated three-pool model, which includes emerging concepts of controls on soil carbon stabilization (e.g., soil physical-chemical protection and priming effect), to constrain projected soil carbon changes in ESMs by the end of this century under different emissions scenarios.
Research sample	Soil incubation experiments across the globe.
Sampling strategy	N/A
Data collection	We used Web of Science and CNKI to search for published papers regarding soil incubation. Data were taken directly from tables or extracted from figures using the GetData (v.2.25) software.
Timing and spatial scale	In total, we gathered 102 peer-reviewed publications covering 178 sites covering eight biomes globally, ranging from arctic permafrost to dry Mediterranean forests.
Data exclusions	We included only soil incubations which satisfied the following criteria: 1) the length of the incubation experiment was longer than six months; 2) the aerobic CO2 production must have been measured over the parallel-warming incubation experiments. If more than one incubated temperature was available, we selected the high-end temperature treatment, as a higher incubation temperature generally leads to a quicker depletion of the fast-cycling carbon pool and therefore enables more information on the slow-cycling carbon decomposability to be retrieved; 3) the initial soil carbon concentration must be available; 4) the soils were incubated without any substrate addition; 5) soil respiration rates in the initial phase must be higher than those at the end of incubation.
Reproducibility	N/A
Randomization	N/A
Blinding	N/A

Did the study involve field work? ☐ Yes ☒ No

Reporting for specific materials, systems and methods

We require information from authors about some types of materials, experimental systems and methods used in many studies. Here, indicate whether each material, system or method listed is relevant to your study. If you are not sure if a list item applies to your research, read the appropriate section before selecting a response.

Materials & experimental systems

n/a	Involved in the study
☒	☐ Antibodies
☒	☐ Eukaryotic cell lines
☒	☐ Palaeontology and archaeology
☒	☐ Animals and other organisms
☒	☐ Clinical data
☒	☐ Dual use research of concern
☒	☐ Plants

Methods

n/a	Involved in the study
☒	☐ ChIP-seq
☒	☐ Flow cytometry
☒	☐ MRI-based neuroimaging

Plants

Seed stocks	N/A
Novel plant genotypes	N/A
Authentication	N/A
